# Nanotechnology-driven strategies for tilapia vaccines: Comparative evaluation of nanoemulsions and silica nanoparticles against *Streptococcus agalactiae*

**DOI:** 10.14202/vetworld.2025.1807-1818

**Published:** 2025-07-08

**Authors:** Angela Mariana Lusiastuti, Siti Nurul Aisyiyah Jenie, Melati Septiyanti, Yulianti Sampora, Tanjung Penataseputro, Thavasimutu Citarasu, Desy Sugiani, Dewi Syahidah, Indah Dwiatmi Dewijanti, Hessy Novita, Tuti Sumiati, Uni Purwaningsih, Suryanto Suryanto, Brata Pantjara, Taufik Hadi Ramli, Pramuanggit Panggih Nugroho, Khairun Nisaa, Annisa Wening Maharani Putri

**Affiliations:** 1 Research Center for Veterinary Science, National Research and Innovation Agency, Jl. Raya Bogor Km. 46 Cibinong, Bogor, 16911, West Java, Indonesia; 2 Research Center for Advanced Chemistry, National Research and Innovation Agency, Jl. Raya Puspiptek 60, Setu, Tangerang Selatan, 15314, Banten, Indonesia; 3 Centre for Marine Science and Technology, Manonmaniam Sundaranar University, Marina Campus, Rajakkamangalam, Kanyakumari District, Tamil Nadu, 629502, India; 4 Research Center for Pharmaceutical Ingredients and Traditional Medicine, National Research and Innovation Agency, Jl. Raya Bogor Km. 46 Cibinong, Bogor, 16911, West Java, Indonesia; 5 Research Center for Fisheries, National Research and Innovation Agency, Jl. Raya Bogor Km. 46 Cibinong, Bogor, 16911, West Java, Indonesia; 6 Department of Aquaculture, The Marine and Fisheries Polytechnic Karawang, The Ministry of Marine Affairs and Fisheries, Jl. Raya Lingkar Tanjungpura-Klari, Karang Pawitan, Karawang Barat, Karawang, 41314, West Java, Indonesia

**Keywords:** fish health, mucosal vaccine, nanoemulsion, nanovaccine, silica nanoparticles, *Streptococcus agalactiae*, tilapia

## Abstract

Streptococcosis, caused by *Streptococcus agalactiae*, is a significant disease in tilapia farming that results in substantial economic losses. While vaccination is the most effective method for prevention, current vaccines face challenges when administered orally or through immersion, primarily due to poor absorption and degradation in the fish’s digestive system. Nanotechnology offers new ways to improve vaccine delivery and effectiveness. This review compares two nanoparticle (NPs)-based systems – nanoemulsions and silica NPs (SiNP) – for delivering vaccines to tilapia. Nanoemulsions are small, stable droplets that protect the vaccine and help it stick to mucosal surfaces, making them more effective in triggering immune responses. SiNP are highly stable and can protect vaccines under harsh conditions but still face challenges in particle size and vaccine loading. The review highlights important factors, including particle size, stability, and surfactant composition, that affect the vaccine’s effectiveness. In practical terms, nanoemulsions are more suitable for use in Indonesia’s tropical aquaculture settings because they are easier to apply, more stable, and more effective in their current formulations. Further research is needed to improve both systems, especially to ensure long-term safety, improve delivery to mucosal tissues, and reduce production costs. Nanotechnology-based vaccines have a strong potential to improve fish health and reduce antibiotic use in aquaculture.

## INTRODUCTION

Streptococcosis, caused by *Streptococcus agala-ctiae*, is a recurring disease in tilapia aquaculture, resulting in substantial economic losses worldwide. Infections with *S. agalactiae* have been documented across nearly all Asian countries, with serotypes Ia, Ib, and III identified as the most prevalent. Notably, serotypes Ia and Ib are frequently associated with high pathogenicity and mortality rates [1, 2]. In Indonesia, outbreaks involving these serotypes have resulted in significant morbidity and mortality among tilapia populations. Similar patterns of disease impact have been observed across Southeast Asia, including in Thailand, Vietnam, Malaysia, and China.

Vaccination is widely regarded as an effective strategy to reduce reliance on antibiotics and miti-gate the emergence of antimicrobial resistance in aqua-culture systems [3]. Over the past two decades, the adoption of fish vaccines has contributed to marked reductions in antibiotic use and disease-related losses. However, challenges remain in achieving consistent long-term protection under field conditions. Available vaccines – monovalent, bivalent, multivalent, and DNA-based – often fail to provide durable immunity due to pathogen variability and host response differences. Moreover, orally administered vaccines are vulnerable to degradation in the gastrointestinal tract, where acidic gastric pH and digestive enzymes can inactivate antigens and impair immune stimulation [3–5].

To address these limitations, nanovaccine technologies have emerged as a promising alternative for delivering sustained and targeted immunological protection. Nanotechnology enables the development of advanced fish vaccines by exploiting the physic-ochemical properties of nanoparticles (NPs), including their high surface reactivity, modifiability, and biocom-patibility [6, 7]. At the nanometer scale, these particles can be engineered to enhance antigen stability, facilitate targeted delivery, and overcome biological barriers. Contemporary nanodelivery systems, such as poly(lactic-co-glycolic acid) (PLGA) [8], chitosan [9], nanoliposomes, and mesoporous silica NPs (MSNs), have demonstrated considerable potential for use in aquaculture as carriers for antigens, proteins, and ther-apeutics [10–13].

Despite significant advances in vaccine develop-ment for aquaculture, the practical deployment of effective mucosal vaccines against *S. agalactiae* in tilapia remains a critical challenge. While injectable vaccines offer strong immunogenicity, they are logistically unsuitable for mass administration, particularly in resource-limited or smallholder systems. Oral and immersion vaccination methods are more feasible for large-scale application but suffer from reduced efficacy due to antigen degradation in the gastrointestinal tract and insufficient mucosal uptake. Although nanotechnology-based approaches have gained attention for their potential to improve vaccine delivery and immunogenicity, there is a limited comparative evaluation of different NPs systems – specifically nanoemulsions and MSNs – in the context of mucosal immunization in tropical aquaculture settings. Most available studies are either exploratory or narrowly focused on single NPs types, lacking comprehensive assessments of their physicochemical properties, immunological performance, practical scalability, and suitability under environmental constraints, such as cold chain instability. Furthermore, few studies have addressed the optimization of formulation parameters, such as particle size, zeta potential, polydispersity index, and surfactant composition in relation to antigen stability and immune system activation in tilapia.

This review aims to critically evaluate and compare the suitability of nanoemulsions and silica NPs (SiNP) as delivery systems for oral and immersion vaccines against *S. agalactiae* in *Oreochromis niloticus*. The review synthesizes existing data on their formulation characteristics, antigen encapsulation efficiency, muc-osal immune activation potential, and application feasibility within tropical aquaculture environments. It also discusses the relevance of NPs physicochemical properties – such as droplet size distribution, stability under variable temperature conditions, and antigen surface interactions – to vaccine efficacy and fish welfare. Emphasis is placed on the specific needs and limitations of tilapia farming in Indonesia and similar regions, including decentralized farming structures, limited cold chain infrastructure, and the need for cost-effective, stress-free vaccination strategies. By addressing present knowledge gaps and practical considerations, this review provides a foundation for guiding future research, optimization strategies, and policy formulation for the development and implementation of nanotechnology-enhanced fish vaccines.

## OVERVIEW OF FISH VACCINATION TECHNIQUES

### Oral, immersion, and injection methods

Fish vaccination is commonly performed thr-ough oral delivery, intraperitoneal or intramuscular injection, or immersion [14]. Vaccines are administered exclusively to healthy fish to ensure an optimal antibody-mediated immune response. In teleost fish, pathogens may enter through external surfaces, such as the skin and gills, as well as through the gastrointestinal tract, making mucosal surfaces critical sites for vaccine uptake. Consequently, oral and immersion methods are particularly suited for stimulating mucosal immune responses. The immune system in teleosts comprises primary lymphoid organs (thymus and head kidney) and secondary lymphoid organs, including the spleen, kidney, and mucosa-associated lymphoid tissue (MALT) [14].

The choice of vaccine administration route depends on several factors, including the nature of the pathogen, the route of infection, the immunological status of the fish, vaccine formulation, cost consid-erations, and the fish’s developmental stage [15]. Although injection-based vaccination is effective, it poses several disadvantages for small-scale tilapia farmers. These include logistical difficulties during grow-out, increased labor demands, and the induction of stress due to handling and injections [16]. Given these limitations and the aquatic environment of fish, oral vaccination remains the most practical and widely adopted approach in aquaculture [17].

### Challenges of oral vaccines

Oral immunization is preferred by fish farmers due to its ease of administration, minimal stress induction, and cost-effectiveness [17]. It contributes to improved fish welfare by eliminating the need for direct han-dling. Nevertheless, its protective efficacy is often lower than that of injectable vaccines. This reduced efficacy arises from inconsistent vaccine intake among fish and the degradation of antigens in the gastrointestinal tract [18, 19].

The acidic environment of the stomach and the enzymatic activity in the intestines can degrade vaccine antigens, reducing their immunogenicity and limiting the stimulation of effective immune responses. Additionally, variability in feed consumption leads to uneven antigen exposure, resulting in inconsistent ant-ibody production and heterogeneous protection within vaccinated populations.

### Barriers to vaccine absorption and mucosal immunity

Ideally, vaccination is administered to fry in hatcheries at around 1 month of age, before transfer to grow-out ponds. In such settings, immersion vaccination is practical, especially when dealing with large fish populations. However, the effectiveness of immersion vaccines depends on several critical parameters, inclu-ding antigen concentration, immersion duration, and water temperature, all of which influence the imm-une response [18].

Compared to injection, immersion vaccination generally results in lower efficacy and shorter duration of protection [20]. This is due to the complex pathway of antigen absorption, which involves passage through mucosal barriers, such as the skin, gills, nasopharynx, and lateral line pores – sites rich in MALT involved in antigen recognition and uptake. For effective imm-une stimulation, vaccine antigens must traverse these mucosal barriers and be processed by antigen-pres-enting cells (APCs), which then present the antigens to the adaptive immune system, initiating antibody production [21]. In addition, physicochemical fac-tors, such as pH and ionic concentration, as well as physiological stress from handling, can further influence antigen uptake and the resulting immune response [18].

## NP SYSTEMS FOR VACCINE DELIVERY

NPs function as advanced delivery vehicles that allow for the controlled and sustained release of vaccine antigens, thereby enhancing therapeutic efficacy. They enable targeted delivery to specific organs, tissues, or cells, increase antigen availability, improve the solubility of hydrophobic compounds, and provide protection against degradation by gastric acid and intes-tinal enzymes during oral administration [6]. These NPs-based systems have been shown to elicit stronger imm-une responses, offering improved protection against infectious agents in fish [22].

Polymeric NPs, such as alginate, chitosan, PLGA, polylactic acid (PLA), dendrimers, and liposomes are commonly applied in vaccine formulations. Conjugating vaccine antigens with nanocarriers is considered an effective strategy to enhance antigen delivery, stim-ulate the immune response, and improve vaccine perfo-rmance [22].

### Polymeric NPs

Polymeric NPs enclosed within biocompatible polymer shells are increasingly recognized for their utility in vaccine delivery. These systems encapsulate antigens within their core, offering protection and controlled release [23]. They exhibit several advantageous prop-erties, including biocompatibility, biodegradability, enhanced stability, and the ability to release antigens in a sustained manner. Notable examples include PLGA, PLA, chitosan, and their copolymers. Polymeric NPs can be categorized into natural and synthetic types. Among them, PLGA is a hydrophobic polymer known for rapid release kinetics and low encapsulation efficiency, which can limit its standalone utility in antigen delivery [23].

#### Natural polymers

Chitosan and alginate are prominent natural polymers utilized in fish vaccine formulations. Chitosan NPs are especially valued for their low molecular weight, mucoadhesiveness, efficient encapsulation, and controlled release properties [24–27]. Their biodegradability, non-toxicity, and compatibility with biological systems make them ideal candidates for fish vaccination [24, 28]. Chitosan-based NPs also stimulate components of the immune system, including natural killer cells, APCs, macrophages, cytokines, and T lymph-ocytes [29]. For instance, Zhang *et al*. [30] demo-nstrated that chitosan NPs induced strong antibody responses against viral infections in carp, further supporting their biocompatibility and efficacy.

#### Synthetic NPs

Synthetic polymers, such as PLGA and PLA are widely used in NPs vaccine development. PLGA, composed of lactic and glycolic acid units, has a tuna-ble degradation rate and adjustable antigen loading capacity based on its monomer ratio [31]. PLA, another biodegradable polymer, degrades into lactic acid, supporting the gradual release of antigens [32]. Both PLGA and PLA offer safe and effective delivery of vaccines, maintaining outer membrane protein functionality and minimizing adverse interactions [23].

### Metal and inorganic NPs

Metal-based NPs – such as those composed of silver, gold, zinc, and titanium – present additional benefits, including controlled particle size, high surface area, efficient loading, and traceability. These NPs exert broad-spectrum antimicrobial effects through non-specific mechanisms, rendering them effective against both Gram-positive and Gram-negative bacteria [33].

MSNs have been employed for delivering antigens, such as dihydrolipoamide dehydrogenase from *Vibrio alginolyticus* in yellow croaker, demonstrating promising results. The release of antigen from MSNs was shown to be pH dependent [34]. In that study, MSNs were coated with hydroxypropyl methylcellulose phthalate HP-55 to protect the immunogen, leading to the induction of both innate and adaptive immunity *in vivo*. MSN-based vaccines are considered highly suitable for oral delivery due to their cost-effectiveness, performance, and ease of administration. In addition, zinc oxide NPs exhibit bactericidal effects against *Aeromonas hydrophila* by disrupting the bacterial membrane and cytoplasm, while also serving a preventative role by inh-ibiting pathogen growth. Titanium dioxide NPs enhance immune responses and act as antibacterial agents in fish [23].

### Liposomes and virus-like particles (VLPs)

Liposomes, another class of NPs used in fish vaccine development, are vesicular carriers composed of hydrophilic and hydrophobic components. Their advantages include non-toxicity, structural mimicry of biological membranes, and ease of biodeg-radation [35, 36]. Liposomal vaccines can take the form of immune-stimulating complexes (ISCOMs) or VLPs. For bacterial vaccines, liposome sizes typically range between 100 nm and 400 nm, whereas ISCOMs for viral vaccines are about 40 nm in size [35].

ISCOMs consist of phospholipids, cholesterol, saponins, and quillaja compounds and are capable of triggering strong cellular immune responses, serving both as carriers and adjuvants to enhance protection [37]. VLPs, composed of capsid proteins, are also effective delivery systems [35]. A patented liposomal vaccine formulation (WO2003101482A2) developed by Keough and Syvert [38] for finfish includes antigenic components associated with liposomes, optionally supplemented with therapeutic agents. This formulation is designed to immunize fish against bacterial and parasitic infections while reducing common adverse effects associated with oil-based vaccines, such as tissue adhesion and pigmentation changes.

### Emulsions

Nanoemulsions represent a widely utilized NPs platform in fish vaccine development. These emulsions exist in two primary forms: Water-in-oil (W/O), typically employed in injectable vaccines, and oil-in-water (O/W), commonly applied in immersion-based vaccination. The droplet size of nanoemulsions ranges from 50 nm to 600 nm. According to Jaiswal *et al*. [39], nanoemulsions are classified into three categories: O/W, where oil is dispersed within a continuous aqueous phase; W/O, where water droplets are suspended in a continuous oil phase; and bi-continuous nanoemulsions. Nanoe-mulsion-based vaccines are capable of delivering antigens to specific target sites, including dendritic cells [39]. However, one of their limitations lies in their thermodynamic instability.

## COMPARATIVE EVALUATION: NANOEMULSION VERSUS NANO-SILICA FOR TILAPIA VACCINE DELIVERY

### Formulation parameters (size, polydispersity index [PDI], zeta potential)

The behavior of bacterial antigens in vaccine formulations is influenced by their Gram-staining characteristics and encapsulation status. Non-enc-apsulated β-hemolytic bacteria, which possess hydro-phobic surface proteins, readily interact with aqueous media and substrates, facilitating more rapid and dense growth. This property also supports impr-oved solubility and stability of antigens. In contrast, encapsulated non-hemolytic bacteria have hydrophilic, carbohydraterich surfaces that hinder adhesion to growth media, leading to slower proliferation. Ther-efore, understanding the surface properties of bacteria is essential for maintaining antigen integrity throughout storage and transport.

As shown in Table 1 [40, 41], antigens in nanoe-mulsions are immobilized within an organic matrix, comprising oleic acid (oil phase) and Tween 80 (surfa-ctant). Conversely, in nanosilica formulations, the particle size of the monovalent and bivalent antigens remains too large, even after maximum sonication, resulting in antigens being located outside rather than within the silica nanopores. Ideally, these antigens should be embedded within the inorganic SiNP matrix.

**Table 1:** The differences between nanoemulsion and nanosilica vaccines

Criteria	Nano-emulsion vaccine	Nanosilica vaccine	References
Formulation parameters			
Size (nm)	Nanoshells (core-shell nanoparticles), protein immobilization in an organic matrix	Nanopore (mesoporous silica nanoparticle), protein immobilization in inorganic matrix	[40]
	Monovalen: 84.5 ± 1.6	Monovalen: 1055.5 ± 26.4	[41]
	Bivalen: 122.7 ± 1.4	Bivalen: 868.6 ± 3.3	
Polydispersity index	Monovalen: 0.377 ± 0.026	Nd	[41]
	Bivalen: 0.410 ± 0.020		
Zeta potential (mV)	Monovalen: −25.3 ± 0.5	Nd	[41]
	Bivalen: −62.7 ± 0.2		
Challenges and limitations	The homogeneity and stability conditions of bivalent vaccines require attention to the same size of each vaccine seed before mixing.	The pore size of the nanosilica must be adjusted to the nanoparticle size of the antigen used.	

*Nd=Not done (the size obtained above 100 nm)

### Protection mechanisms

Upon immersion vaccination, MALT is the first to respond to antigen exposure. This response is sequentially mediated by skin-associated lymphoid tissue, gill-associated lymphoid tissue, nasopharynx-associated lymphoid tissue (NALT), buccal mucosa, and gut-associated lymphoid tissue (GALT). The immersion process stimulates the secretion of mucus containing various bioactive molecules, such as antibodies, agglu-tinins, hemolysins, complement proteins, lectins, lysoz-yme, C-reactive proteins, proteases, antimicrobial peptides, and enzymes, which act to inhibit pathogen entry and replication. The effectiveness of immersion vaccines depends on achieving the correct exposure time and dosage during administration [42].

Both nanoemulsion and nanosilica formulations have demonstrated improved antigen adhesion and uptake compared to unencapsulated vaccines, thereby enhancing the efficacy of the immune response during immersion. Oral vaccination primarily targets mucosal lymphoid tissues, including NALT, buccal mucosa, pharyngeal mucosa, and GALT [43–46]. MALT not only facilitates innate and adaptive immune responses through lymphocytes, macrophages, granulocytes, and eosinophils, but also contributes to the systemic distribution of B and T cells, which is necessary for robust mucosal immunity.

### Application feasibility (oral vs. immersion)

In Indonesia, the tropical climate and widespread geography present logistical challenges for vaccine storage and distribution. Vaccines are sensitive biolo-gical products that can degrade when exposed to sunlight or fluctuating temperatures. Maintaining an unbroken cold chain (2°C–8°C for freeze-sensitive vaccines and 15°C–25°C for heat-sensitive vaccines) is therefore essential.

To ensure vaccine stability during transport, alternative preservation strategies are recommended, including (1) lyophilization with cryoprotectants, such as sucrose and trehalose, to obtain a dry vaccine form and (2) antigen immobilization within organic or inorganic matrices using covalent or non-covalent bonding [47]. Nano-silica (SiO_2_NPs) has been shown to improve vaccine stability under harsh environmental conditions [48, 49].

In aquaculture, a variety of NPs systems have been employed, including nanoliposomes [49], macro-molecular NPs [50], inorganic NPs [51], and ISCOMs [52]. Among these, inorganic NPs are often preferred due to their superior physicochemical properties compared to organic carriers [53, 54]. In Indonesia, natural polymeric NPs, such as chitosan, hyaluronic acid, and alginate, are used in oral fish vaccines. These materials are biodegradable, biocompatible, and capable of binding antigens effectively [55]. Their nanoscale dimensions enable sustained antigen release and enhanced resistance to gastrointestinal degradation, thereby promoting better antigen uptake by APCs and higher antibody production. Synthetic polymers, such as PLGA and PLA, are also under investigation for oral vaccine delivery [56–58].

In nanoemulsion systems, surfactants play a crucial role in stabilizing oil and water phases. Their small droplet sizes allow for the effective encapsulation of both hydrophilic and hydrophobic antigens – hydrophilic antigens are distributed in the aqueous phase, while hydrophobic antigens integrate into the oil phase. The nanoscale dimensions enable controlled antigen release, thereby facilitating prolonged immune stimulation in vaccinated fish. Lusiastuti *et al*. [41] developed and analyzed both nanoemulsion and nanosilica-based vaccines. As reported in Fredriksen and Grip [59], nanoemulsion droplets range from 50 nm to 600 nm, whereas inorganic NPs, such as nanosilica range from 2 nm to 1,000 nm.

**Figure 1:**
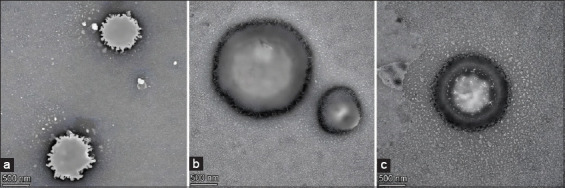
Transmission electron microscope - Nanoemulsion fish vaccine: (a) monovalent *Streptococcus agalactiae* vaccine with nanoemulsion, (b) bivalent *S. agalactiae* and *Aeromonas hydrophila* vaccine with nanoemulsion, and (c) vaccine without nanoemulsion (using single particle analysis Tundra 100kV) [41].

In the study by Lusiastuti *et al*. [41], 500 nm particles were used for *S. agalactiae* and *A. hydrophila*. Their results demonstrated variations in droplet size, PDI, and zeta potential for monovalent and bivalent formulations (Table 2) [41], reflecting the influence of bacterial characteristics on NPs behavior. Transmission electron microscopy (Tundra 100 kV) revealed that in nanoemulsion-based vaccines, bacterial antigens were encapsulated within the emulsion droplets (Figures 1a and b) [41], unlike in the conventional vacc-ine, where antigens remained uncoated (Figure 1c).

**Table 2:** Droplet vaccine, polydispersity index, and zeta potential of monovalent and bivalent vaccine Lusiastuti *et al.* [41].

Vaccine treatment	Particle size (nm)	Polydispersity index	Zeta potential (mV)
Vaccine (control)	105.3 ± 0.1	0.592 ± 0.054	−58.2 ± 0.3
Monovalent vaccine (*S. agalactiae*) 100% + Tween 80	84.5 ± 1.6	0.377 ± 0.026	−25.3 ± 0.5
Bivalent vaccine (*S. agalactiae* and *A. hydrophila*) 100% + Tween 80	122.7 ± 1.4	0.410 ± 0.020	−62.7 ± 0.2

*S. agalactiae=Streptococcus agalactiae, A. hydrophila=Aeromonas hydrophila*

**Figure 2:**
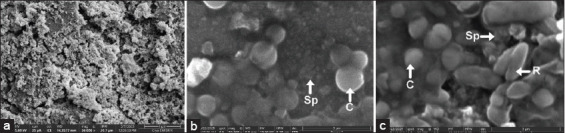
Scanning electron microscope (SEM) - Nano-silica fish vaccine: (a) Silica particles without vaccine, (b) silica particles mix with monovalent vaccine of *Streptococcus agalactiae* (coccus shape), and (c) silica particles mix with bivalent vaccine of *S. agalactiae* (coccus) and *Aeromonas hydrophila* (rod with rounded tip). C=Coccus, R=Rods, Sp=Silica particles. (Using Cryo field emission-SEM Aquilos 2) [41].

Scanning electron microscopy (Figure 2) [41] indicated that in nanosilica-based vaccines, the silica particles remained outside the much larger bacterial antigens, failing to encapsulate them properly. Both vaccine types offer the advantages of needle-free application, low fish stress, and targeted antigen delivery to mucosal tissues, eliciting both systemic and local immune responses. Therefore, mucosal vaccines hold significant practical and immunological advantages.

Oral vaccines, however, face degradation challen-ges in complex feed matrices and within the digestive system. Enzymatic breakdown reduces antigen delivery to immune cells, thereby hindering antibody production. NPs improve oral vaccine performance by stabilizing antigens on their surfaces [60]. Nano-silica, with its high surface area and mesoporous architecture, is well suited for antigen loading. Its porosity and surface chemistry enhance antigen retention and delivery efficiency. Mesoporous nano-silica has demonstrated excellent biocompatibility, non-toxicity, and thermal resistance up to 1,500°C, with abundant hydroxyl groups enabling broader functionalization [61–64].

For nanocarriers to be effective, they must possess optimal cargo-loading capacity, consistent particle size and shape, and robust biocompatibility and stability. SiNP are preferred for their ease of synthesis, adjustable size, and stability. However, in the study by Lusiastuti *et al*. [41], nano-silica conjugation with monovalent and bivalent vaccine antigens produced particles that were still too large (Table 3) [41], reducing their efficacy in targeted antigen delivery.

**Table 3:** SOR of fish vaccine using nanoemulsion formula Lusiastuti *et al*. [41].

Composition	SOR 5%	SOR 10%	SOR 15%	SOR 20%
Fish vaccine	30	30	30	30
Oleic acid	66.5	63	59.5	56
Surfactant	3.5	7	10.5	14
• Span 80 (10%)		0.7	1.05	1.4
• ween 80 (90%)	3.15	6.3	9.45	12.6

SOR=Surfactant/oil ratio

The particle sizes obtained (Table 3) approached 2 micrometers comparable to the dimensions of bacterial antigens – making them suboptimal for intracellular delivery. Thus, reformulating nanosilica to achieve smaller, more functional particle sizes is necessary to enhance vaccine efficacy and stability. Present conju-gation techniques rely primarily on non-covalent interactions, including hydrogen bonding, electrostatic forces, hydrophobic interactions, ionic bonding, and van der Waals forces, all of which are relatively weak and reversible [65, 66].

## MATERIALS AND PRACTICAL CONSIDERATIONS IN INDONESIA

### Challenges in vaccine cold chain

Vaccine potency can be compromised by temperature instability during storage and trans-portation. Malfunctions in refrigeration units, accidental freezing or thawing, and the use of inappropriate transport containers frequently lead to structural damage to antigens. In addition, exposure to chemical contaminants (e.g., peroxides and metals), light, and fluctuations in pH and temperature can further reduce vaccine shelf life. These stressors often result in repeated freeze–thaw cycles, which can lead to antigen denaturation. This process exposes hydrophobic regions that facilitate aggregation with adjacent antigens, forming large, insoluble complexes that ultimately impair vaccine stability and immunogenic performance [67].

Nanoemulsions, consisting of an aqueous and an oil phase stabilized by emulsifiers and co-emulsifiers, are biphasic systems suitable for vaccine delivery [68]. Several key parameters influence the efficacy of nanoemulsified vaccines, including the surface area of antigens, their uniform dispersion within the emulsion, and the capacity for sustained and controlled antigen release [69]. In addition, vaccine performance is signifi-cantly affected by factors such as nanoemulsion stability, droplet size, carrier type, and PDI [70].

In practical applications, immersion is the preferred method for administering vaccines to fry. After 15–30 min of immersion, the fry is transferred to grow-out ponds. Vaccines formulated for immersion are typically O/W nanoemulsions, where water serves as the continuous phase. If oil droplets are surrounded by water molecules, the system is categorized as a W/O emulsion; alternatively, if water acts as the bulk phase surrounding dispersed oil droplets, it functions as a diluent [71]. Increasing the water content in O/W emulsions reduces the density of NPs, thereby modu-lating the intensity of the immune response [71]. Antigens suspended in the aqueous bulk phase are efficiently transported and adsorbed onto NPs surfaces, enabling rapid recognition by APCs and subsequent activation of B cells to initiate antibody production [71].

### Local adaptation: Surfactants, nanoemulsion stabilizers, and nanosilica matrix use

**Figure 3:**
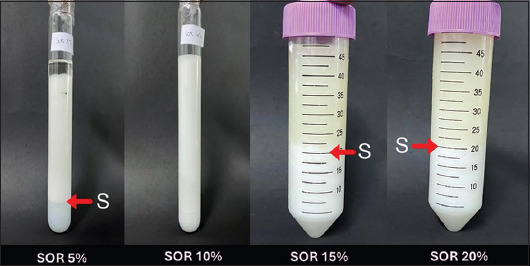
Surfactant/oil ratio (SOR) optimization of nanoemulsion fish vaccine: SOR (5%, 10%, 15%, and 20%) in bacterin of fish vaccine, and SOR 10% is the best; there is no separation (s) and stable emulsion than SOR 5%, 15%, and SOR 20% [41].

Lusiastuti *et al*. [41] conducted optimization studies on the surfactant-to-oil ratio (SOR) to identify an effective nanoemulsion formulation, as shown in Table 3. Four SOR levels – 5%, 10%, 15%, and 20% – were tested. Surfactants with varying hydrophilic-lipophilic balance (HLB) values were incorporated into the outer phase of the emulsion to form either O/W or W/O emulsions [71]. The oil phase was composed of oleic acid (HLB 17), Tween 80 (HLB 15), and Span 80 (HLB 4.3), while the aqueous phase contained the vaccine antigens. The 10% SOR formulation produced the most favorable results, as evidenced by its reduced phase separation (Figure 3) [41]. An HLB value >10 was selected to promote the formation of O/W emulsions suitable for oral or immersion vaccination.

Figure 3 demonstrates that the SOR plays a critical role in determining particle density and, consequently, the initiation of immune responses. The water content of each formulation affects the resulting particle density, which in turn influences antigen adsorption rates [72]. These rates are modulated by hydrated ions, which facilitate the interaction between antigens and APCs. Environmental pH also plays a role; acidic conditions (pH 4–5) promote the aggregation of nanoemulsion droplets, whereas alkaline conditions contribute to droplet stabilization [72]. Larger droplets can form due to insufficient surfactant concentration, resulting in incomplete dissolution of the oil phase. Conversely, high surfactant levels can lead to the formation of smaller droplets by achieving a phase inversion point where the oil becomes completely solubilized [72]. At the appropriate phase transition, the surfactant and oil components align into a layered structure, which influences critical vaccine properties, such as stability and PDI [72].

NPs in the range of 200–600 nm are recognized by APCs and can trigger a Th1-type cellular immune response. In contrast, particles measuring 2–8 μm tend to stimulate humoral immunity through macrophage-mediated phagocytosis [73]. A threshold of 500 nm serves as a critical point – particles smaller than 500 nm primarily stimulate cellular responses, while those larger than 500 nm favor humoral responses [73].

Uniform particle size distribution is crucial for the effective formulation of nanoemulsion-based vaccines. Inconsistencies in size may result in collisions between particles, promoting aggregation and reducing stability [74]. Consequently, the PDI is a vital parameter for evaluating the stability and efficacy of nanoemulsions [75].

As shown in Table 4 [41], the conjugation of monovalent and bivalent vaccines with nanosilica resulted in particle sizes exceeding 600 nm, dimensions that exceed optimal recognition thresholds for T-helper cells. During the sonication process, bacterial antigens were observed to range from nm 400 to 500 nm. According to Danaei *et al*. [76], particles with a PDI >0.5 are prone to collisions and aggregation, especially when interacting with nanosilica, often resulting in particle sizes surpassing 600 nm, as documented in Table 4.

**Table 4:** Conjugation of fish vaccine with nano-silica resulting in large particle size Lusiastuti *et al*. [41].

No.	Nano-silica conjugated with fish vaccine	Particle size (nm)*
1.	Nano-silica + Monovalent vaccine 1:1	1669.6 ± 204.2
2.	Nano-silica + Monovalent vaccine 1:2.5	1583.5 ± 45.3
3.	Nano-silica + Monovalent vaccine 1:5	1055.5 ± 26.4
4.	Nano-silica + Bivalent vaccine 1:1	785.2 ± 70.0
5.	Nano-silica + Bivalent vaccine 1:2.5	868.6 ± 3.3
6.	Nano-silica + Bivalent vaccine 1:5	1296.2 ± 56.8

* Using dynamic light scattering (Horiba SZ 100)

## PROPOSED OPTIMIZATION STRATEGIES

In light of the growing challenges associated with climate change and global food security, the development of effective nanovaccines for aquaculture holds substantial strategic importance. To fully harness their potential, well-defined optimization strategies are necessary.

### Modulation of NPs properties

There remains a critical need for the continued refinement of NPs formulations to ensure the production of non-aggregating, physically and chemically stable particles. These NPs must exhibit favorable biodist-ribution profiles and engage in predictable, targeted interactions within biological systems to maximize their efficacy and safety.

### Chronic exposure and biosafety studies

Comprehensive evaluations of the chronic exposure effects of nanosilica and nanoemulsions are essential, particularly concerning their long-term toxic-ological impacts *in vivo* using established fish models. Robust safety assessments should be conducted across different fish species and developmental stages to ensure cross-species compatibility. In addition, research into NPs-microbiota interactions is needed to uncover any unforeseen consequences on host health and immune regulation. Before large-scale implementation, thorough risk assessments must be conducted to add-ress environmental dissemination and the potential for bioaccumulation of these nanomaterials.

### Formulation for mucosal delivery enhancement

Enhancing vaccine efficacy through improved mucosal delivery requires targeted efforts to reduce NPs aggregation and promote site-specific delivery to mucosal tissues. Refining the design of NPs to improve antigen uptake and stimulate localized immune respo-nses is a key step toward broader implementation. Future research should focus on developing scalable, economically viable nanovaccine platforms that support the widespread integration of aquaculture immun-ization programs.

## CONCLUSION

This review delineates the comparative potential of nanoemulsions and SiNP as innovative delivery platforms for tilapia vaccines targeting *S. agalactiae*, emphasizing their respective physicochemical charact-eristics, antigen-encapsulation efficacy, and field adap-tability. Nanoemulsions emerged as a more promising system under tropical aquaculture settings, such as Indonesia, offering greater formulation stability, effective antigen delivery to mucosal surfaces, and practical ease of application through immersion or oral routes. In contrast, while SiNP exhibits superior environmental resilience and structural integrity, challenges related to particle size and inefficient antigen encapsulation currently hinder their field applicability.

The implementation of nanovaccine platforms has the potential to transform preventive health management in aquaculture by enabling non-invasive immunization strategies that reduce fish stress and labor costs. Nanoemulsion-based vaccines, particularly those optimized for mucosal delivery, are especially advantageous for decentralized and small-scale aqua-culture systems due to their low cold-chain dependency and enhanced immunogenicity.

The strengths of nanotechnology-driven vaccines lie in their customizable surface properties, controlled release kinetics, improved antigen protection, and ability to target mucosal immunity. In addition, their compatibility with lyophilization and scalability offers a practical edge for mass production and transport, particularly in low- and middle-income aquaculture-producing countries.

Despite these advantages, key limitations remain. These include aggregation tendencies of NPs, insufficient encapsulation efficiency in silica-based systems, the lack of extensive *in vivo* biosafety data across multiple fish species, and the high production cost of nanocarriers relative to conventional vaccines. Furthermore, present formulation techniques require refinement to ensure consistency in particle size and zeta potential, which directly influence immunogenic performance.

Future research must focus on the development of next-generation NPs formulations with optimized surface chemistry and functional group density to enhance antigen adherence and uptake. In parallel, chronic toxicity, environmental fate, and NPs-microbiota interactions must be thoroughly investigated to estab-lish comprehensive biosafety profiles. Moreover, trans-lational studies – including field trials and cost-benefit analyses – are essential for guiding commercial scale-up and regulatory approval pathways.

Nanovaccine technologies hold immense promise for redefining fish health management by providing sustainable, effective, and farmer-friendly alternatives to the use of antibiotics. Among the systems explored, nanoemulsions currently offer the most viable path for field deployment in tilapia farming. Nonetheless, continued interdisciplinary collaboration and techno-logical refinement are imperative to bridge existing knowledge gaps and to ensure that nanotechnology reaches its full potential as a cornerstone of sustainable aquaculture immunoprophylaxis.

## AUTHORS’ CONTRIBUTIONS

AML: Investigation, formal analysis, data analysis, data curation, and drafted the manuscript. SNAJ, MS, YS, TP, BP, and DS: Investigation, data analysis, methodology, and data curation. TC and SS: Validation and edited the manuscript. DSy, IDD, HN, TS, UP, THR, PPN, KN, and AWMP: Data collection and formal analysis. All authors have read and approved the final manuscript.
